# Clinical Relevance of Mortalin in Ovarian Cancer Patients

**DOI:** 10.3390/cells12050701

**Published:** 2023-02-23

**Authors:** Alicja Rajtak, Arkadiusz Czerwonka, Michael Pitter, Jan Kotarski, Karolina Okła

**Affiliations:** 1 The First Department of Oncologic Gynecology and Gynecology, Medical University of Lublin, 20-081 Lublin, Poland; 2 Department of Biochemistry and Molecular Biology, Medical University of Lublin, 20-081 Lublin, Poland; 3 Department of Surgery, University of Michigan, Ann Arbor, MI 48109-2200, USA

**Keywords:** mortalin/mtHsp70/GRP75/PBP74/HSPA9/HSPA9B, ovarian cancer, biomarker, metastasis, recurrence, OXPHOS, EMT, stemness, RNAseq

## Abstract

**Background**: Ovarian cancer (OC) is the most lethal malignancy of the female reproductive tract. Consequently, a better understanding of the malignant features in OC is pertinent. Mortalin (mtHsp70/GRP75/PBP74/HSPA9/HSPA9B) promotes cancer development, progression, metastasis, and recurrence. Yet, there is no parallel evaluation and clinical relevance of mortalin in the peripheral and local tumor ecosystem in OC patients. **Methods**: A cohort of 92 pretreatment women was recruited, including 50 OC patients, 14 patients with benign ovarian tumors, and 28 healthy women. Blood plasma and ascites fluid-soluble mortalin concentrations were measured by ELISA. Mortalin protein levels in tissues and OC cells were analyzed using proteomic datasets. The gene expression profile of mortalin in ovarian tissues was evaluated through the analysis of RNAseq data. Kaplan–Meier analysis was used to demonstrate the prognostic relevance of mortalin. **Results**: First, we found upregulation of local mortalin in two different ecosystems, i.e., ascites and tumor tissues in human OC compared to control groups. Second, abundance expression of local tumor mortalin is associated with cancer-driven signaling pathways and worse clinical outcome. Third, high mortalin level in tumor tissues, but not in the blood plasma or ascites fluid, predicts worse patient prognosis. **Conclusions**: Our findings demonstrate a previously unknown mortalin profile in peripheral and local tumor ecosystem and its clinical relevance in OC. These novel findings may serve clinicians and investigators in the development of biomarker-based targeted therapeutics and immunotherapies.

## 1. Introduction

Ovarian cancer (OC) is the most lethal of all gynecological malignancies [1]. Specifically, 75% of patients are diagnosed at advanced stages, and 75% of these patients die within 5 years. The majority of these mortalities are due to recurrence of disease, resistance to current therapies, significant heterogeneity of tumors, and immune suppression in tumor microenvironments (TMEs) [2,3,4,5]. Additionally, early-stage OC is usually asymptomatic; therefore, it is mainly diagnosed at an advanced stage, during spread of disease across the peritoneal cavity, usually accompanied by malignant ascites [6]. While in the initial phase of the disease, OC patients usually respond well to cytoreductive debulking surgery and chemotherapy, bur most women develop recurrence of a chemotherapy-resistant form of the disease within 12 to 18 months [7,8]. In recent years, immunotherapy has revolutionized cancer treatment; however, the results of immunotherapy are unsatisfactory in OC [9]. Although OC is an immunogenic tumor that can be recognized by the host immune system, spontaneous antitumor immune response has only been demonstrated in about 50% of patients, mainly due to tumor-favorable immunosuppressive TMEs [10]. The previous results of our and other research groups described the establishment of the immunosuppressive milieu in OC, including the presence of monocytic myeloid-derived suppressor cells (M-MDSCs) and others [4,11,12]. This provides a very rich “soil” in TMEs for immune escape of cancer cells. Currently, there are no approved immunotherapies for OC. However, many researchers are looking for ways to control OC and to enhance antitumor immunity as a means to increase the patient’s responsiveness to (immuno)therapy.

Mortalin (mtHsp70/GRP75/PBP74/HSPA9/HSPA9B) is a member of the heat-shock protein (Hsp) 70 family of chaperone proteins which regulates physiological functions of cells such as controlling oxidative stress response of cells, mitochondrial function, and maintaining physiological balance [13]. Overexpression of mortalin can play an essential role in cancer including the regulation of cell proliferation, progression, metastasis, apoptosis, and phenotype of cancer stem cells. Additionally, mortalin has been found to promote chemotherapy resistance [14]. Interestingly, mortalin can also regulate proinflammatory cytokines and immune response. Indeed, immunoregulatory properties of these proteins have led to their classification as “chaperokines” [15]. Consequently, broad evidence related to mortalin-related promotion of carcinogenesis suggests this factor as a promising strategy for anticancer (immuno)therapy. Mortalin is constitutively expressed in eukaryotes at low levels in physiological conditions [16]; however, it is often upregulated in human cancers including breast, colon, lung, pancreatic, cancers, and hepatocellular carcinoma (HCC) [17,18,19,20,21]. Clinically, the abnormal expression of mortalin has been shown to be a poor prognostic biomarker in breast, lung, colorectal, and pancreatic cancers and glioblastoma [14].

Although previous studies reported mortalin within the tumor tissue of OC, a paucity of data on soluble mortalin level in the peripheral and local ecosystems of OC exist. Moreover, results derived from the expression of mortalin in tumor tissue are conflicting. Whereas some authors have shown elevated expression level of mortalin in OC [22], another study revealed no changes in the level of mortalin expression versus control [13]. Extracellular soluble blood mortalin was documented in colorectal cancers [17,23]; however, soluble mortalin data are lacking in OC.

## 2. Materials and Methods

### 2.1. Patients Characteristic and Sample Collection

Fresh whole peripheral blood and ascites samples were obtained before or during surgery in First Department of Oncologic Gynecology and Gynecology, Medical University of Lublin. Whole blood and/or ascites samples were collected from patients suffering from 50 OC and 14 benign ovarian tumors. Blood samples were also collected from 28 healthy women. The inclusion criterion for patients was diagnosis of ovarian pathology (OC, benign). Exclusion criteria were an age <18, history of previous cancers, chemo- or radiotherapy prior to surgical procedure, and presence of allergic, autoimmune disorders, infections. Clinical data from ovarian pathology, i.e., benign and OC patients, are presented in Table 1. Kurman–Shih classification was used for determined of OC type [24]. The clinical data of patients, i.e., International Federation of Gynecology and Obstetrics (FIGO) stage, grade, histology, and treatment history, were obtained from a centralized database. Written consent was obtained from participants. Ethical approval was granted by our Institutional Ethics Committee.

### 2.2. Sample Preparation

Pretreatment, fresh, venous whole peripheral blood (9 mL) was collected into heparinized tubes (Sarstedt, Nümbrecht, Germany) before surgery. Fresh ascites samples were obtained aseptically during the operation. Cell-free blood plasma and ascites fluid samples were obtained using centrifugation (2000 rpm/10 min). Blood plasma, ascites fluid, and tumor tissue samples were stored at −80 °C before testing.

### 2.3. Soluble Mortalin Measurement

Blood plasma and ascites fluid samples were blinded for the contractor who performed experiments. Mortalin concentrations (pg/mL) were measured using an enzyme-linked immunosorbent assay (ELISA, Human Mortalin (75 kDa glucose-regulated protein) ELISA Kit, FineTest, Wuhan, China) according to the manufacturer’s protocol (detection range: 15.625–1000 pg/mL and sensitivity: <9.375 pg/mL). Samples were measured in duplicates, and the coefficient of variance (%CV) was <20%. Absorbances were measured using an ELX-800 plate reader and analyzed by KC Junior software (Bio-Tek, Instruments, Winooski, VT, USA).

### 2.4. Isolation of Cells and Flow Cytometry

Mononuclear cells (MCs) were isolated from OC blood and ascites samples as we previously described [5]. In brief, blood and ascites specimens were centrifuged (1500 rpm/10 min), and MCs were isolated by density gradient centrifugation. Next, isolated MCs were collected, washed, and cryopreserved until analysis. To determine the frequency of blood-circulating and ascites-infiltrating HLA-DR^−/low^CD14^+^ M-MDSCs among isolated MCs, cell suspensions were stained for 30 min using monoclonal antibodies including CD14-PE-Cy7 (clone: M5E2, Catalog No. 557742) and HLA-DR-PerCP-Cy5.5 (clone: G46-6, Catalog No. 560652) (all obtained from BD Biosciences, Franklin Lakes, NJ, USA). Flow cytometric data were collected using BD FACSCanto Flow Cytometer (BD Biosciences, Franklin Lakes, NJ, USA) and analyzed using FCS Express 6 Flow Cytometry (De Novo Software, Pasadena, CA, USA). An EasySep™ Human EpCAM Positive Selection Kit II (StemCell) was used for isolation of tumor cells. An EasySep™ Human CD14 Positive Selection Kit II (Stem cell) was used for isolation of tumor-infiltrating myeloid cells.

### 2.5. Mortalin Protein Analysis

The Clinical Proteomic Tumor Analysis Consortium (CPTAC) [25] generated the mass spectrometry-based proteomic data used in this publication. Analysis was performed using the University of Alabama at Birmingham Cancer (UALCAN) data analysis portal with data of normal tissues (*n* = 25) and OC primary tumors (*n* = 100). For validation of mortalin protein expression, 10 normal tissue samples which were matched with tumor samples from the same HGSOC patient were used from proteomic dataset [26]. Mortalin expression of a matched cell-line series from three patients with high-grade serous OC (HGSOC) before and after development of clinical platinum resistance (PEA1/PEA2, PEO14/PEO23) was analyzed using the Ovarian Cancer Cell Line Data Portal [27]. HSPA9 polyclonal antibody (Invitrogen) was used for analysis of protein in tumor and myeloid cells from HGSOC tissues using Western blot analysis.

### 2.6. Bioinformatics Analysis

Single-cell RNA-seq counts were obtained from the Gene Expression Omnibus database with the accession numbers GSE211956 (eight HGSOC patients) and GSE130000 (eight OC samples, including four primary tumors, two peritoneal metastases, and two relapse tumors). The UMI counts, gene information, and barcode matrix output from the Cell Ranger software pipeline provided by 10x Genomics were used for downstream analysis with the pipeline of Seurat (version 4.1.0, Satija Lab, Cambridge, MA, USA) R (version 4.2.0, Satija Lab, Cambridge, MA, USA). For data quality control, cells with fewer than 200 genes detected and cells with greater than 30% mitochondrial RNA content were excluded from analysis. Following this step, 20,096 cells passed these filters and were included in downstream analysis. Counts on the filtered matrix of each gene were then normalized with the total library size with the Seurat ‘NormalizeData’ function. To detect the biologically meaningful variation across cells, we used a subset of highly variable genes (2000) identified by the function of ‘FindVariableGenes’ from Seurat to perform unsupervised clustering. Next, using the ‘ScaleData’ function, we applied a linear transformation which shifts the expression of each gene so that the mean expression across cells is 0 and scales the expression of each gene so that the variance across cells is 1. This gives equal weight to each gene so that highly expressed genes do not dominate. Linear dimensionality reduction (PCA) was performed using the function ‘RunPCA’. To partition the cellular distance matrix into clusters, the graph-based ‘FindClusters’ function was used with the resolution set to 0.9. Next, UMAP projections were generated to visualize the clusters of cells localized in the graph-based clusters using the ‘RunUMAP’ function with the same principal components described above. Cluster markers were identified by finding differentially expressed genes between cells in a single cluster versus all cells in all other clusters using the ‘FindAllMarkers’ function (Seurat). To find statistical associations between human ovarian tumor-derived *HSPA9* and other protumor gene expression, we calculated Pearson correlation coefficients (R) and *p*-values between *HSPA9* and other key gene expression vectors each containing the expression values of the gene of interest per cell. Next, we separated the ovarian tumor cells according to low and high expression of *HSPA9* on the basis of the normalized mean expression and then conducted gene set enrichment analysis (GSEA) on the ovarian tumor cells with high expression of *HSPA9* using the ‘gseGO’ function of the clusterProfiler (3.18.1, Yu G, Guangzhou, China) R package. The GSEA determined whether genes associated with high expression of *HSPA9* were together involved in key cellular pathways in cancer progression. Gene sets were obtained from MSigDB, Gene Ontology and Disease Ontology databases. The bulk RNA-seq dataset [28] for malignant fluids of serous OC, including tumor-derived organoids and malignant effusion cells (no cultured) paired with normal ovarian tissues, was used for the HSPA9 expression profile.

### 2.7. Kaplan–Meier Analysis

For in silico analysis, the Kaplan–Meier plotter database was used to further analyze the prognostic relevance of mortalin gene expression using gene chip data in serous OC [29]. Only the optimal probe set (JetSet best probe set) for *HSPA9* was used. The probabilities of OS of the study group were estimated using the Kaplan–Meier method (Mantel–Cox log-rank test). OS was defined as the time from the primary diagnosis until death.

### 2.8. Statistical Analysis

All data were performed using GraphPad Prism 8.0 (La Jolla, CA, USA) and analyzed using the Mann–Whitney U test, one-way analysis of variance (ANOVA), and *t*-test. Spearman correlation was used for calculation of correlation coefficient r between parameters. A *p* -value < 0.05 was considered significant (*p* < 0.05 *, *p* < 0.01 **, *p* < 0.001 ***, *p* < 0.0001 ****). For prognostic relevance of *HSPA9* mRNA, the Kaplan–Meier plotter tool was used (http://kmplot.com/analysis/ (accessed on 15 December 2022)) [29].

## 3. Results

### 3.1. Mortalin Is Upregulated in Local Tumor Environments in Ovarian Cancer Patients

**Figure 1:**
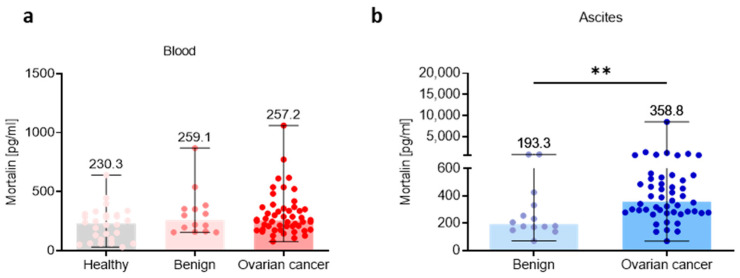
Mortalin profile in the peripheral blood and local ascites tumor environments. Soluble mortalin levels were measured (**a**) in the blood plasma from cohorts of healthy, benign ovarian tumors patients, and ovarian cancer patients, and (**b**) in the ascites fluid from cohorts of benign ovarian tumors patients, and ovarian cancer patients. Bars represent medians and ranges. Significant differences between study groups are marked using asterisks: ** *p* < 0.01.

To study the mortalin profile in the three different ecosystems, we analyzed its level in the blood plasma, ascites fluid, and tumor tissue. We determined the level of extracellular soluble mortalin in healthy women, benign ovarian tumors, and OC using ELISA. Due to current inability to obtain normal ascites fluid, we used ascites from patients with benign ovarian tumors to compare mortalin concentrations in benign and malignant ascites fluid (ELISA). When we analyzed the level of mortalin in the blood plasma, we found no significant change of this factor in OC patients compared to healthy women and benign ovarian tumors (*p* > 0.05; Figure 1a). In contrast, in the ascites fluid, we noted a significantly higher level of this protein in OC patients versus patients with benign ovarian tumors (*p* < 0.01; Figure 1b). In concordance with this, analysis of mortalin in CPTAC-generated mass spectrometry-based proteomic data revealed significantly higher levels of mortalin in OC primary tumors compared to nontumor tissues (*p* < 0.001) (Figure S1a). Similarly, data showed lower mortalin protein expression in normal tissues compared to adjacent tumor tissues in HGSOC (*p* < 0.01) (Figure S1b).

### 3.2. Mortalin Levels Are Higher in Local Ascites Tumor Environment Compared with Adjacent Peripheral Blood Environment

**Figure 2:**
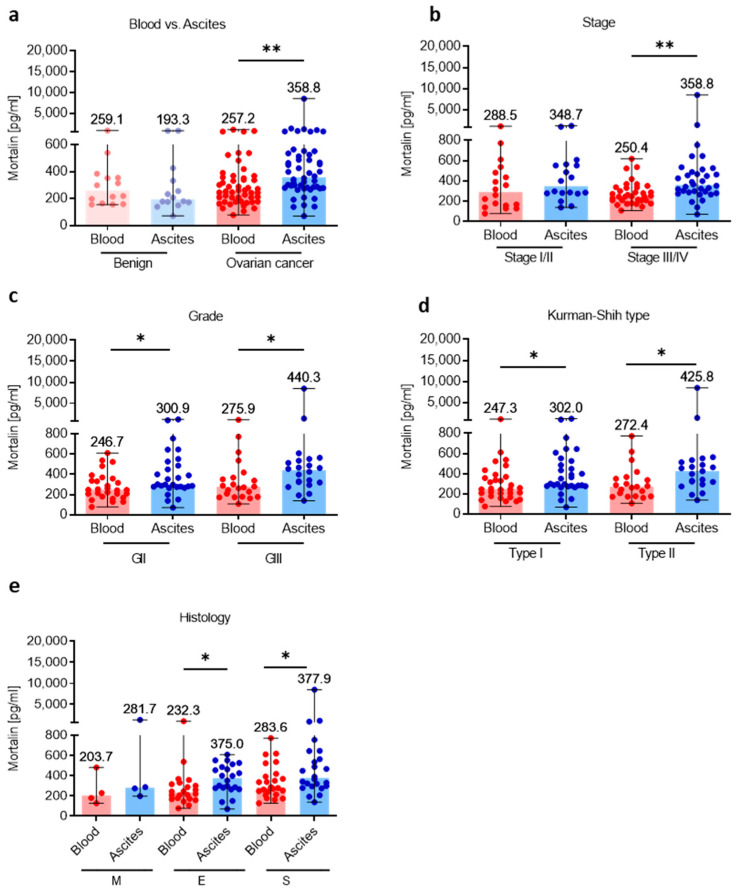
Comparative analysis of mortalin in the blood plasma and accompanying ascites fluid. The levels of mortalin in the blood plasma and ascites fluid from patients with ovarian cancer and benign ovarian tumors (**a**). The levels of blood plasma and ascites fluid mortalin in ovarian cancer patients with different clinicopathologic characteristics, i.e., stage (**b**), grade (**c**), Kurman–Shih type (**d**), and histology type (**e**). Bars represent medians and ranges. * *p* < 0.05, ** *p* < 0.01. E—endometrioid OC; M—mucinous OC; S—serous OC.

Having shown high levels of soluble mortalin in the ascites fluid in OC patients, we comparatively analyzed mortalin concentrations using paired samples of blood and ascites and asked questions about the level of mortalin in these two ecosystems. As we surmised, we showed higher abundance of mortalin in the ascites fluid versus blood plasma in OC, but not in the benign tumors (*p* < 0.01; *p* > 0.05, respectively, Figure 2a). Similarly, when we compared mortalin level in the blood plasma and ascites fluid in patients with different clinicopathologic features, we revealed higher accumulation of ascites fluid mortalin in advanced stage (stage III and stage IV, *p* < 0.01; Figure 2b), both grades (GII and GIII, *p* < 0.05; Figure 2c), both Kurman–Shih types (type I and type II, *p* < 0.05; Figure 2d), and both endometrioid and serous types of OC (both *p* < 0.05; Figure 2e) compared to blood mortalin.

### 3.3. Association of Mortalin Levels with Clinicopathological Features

**Figure 3:**
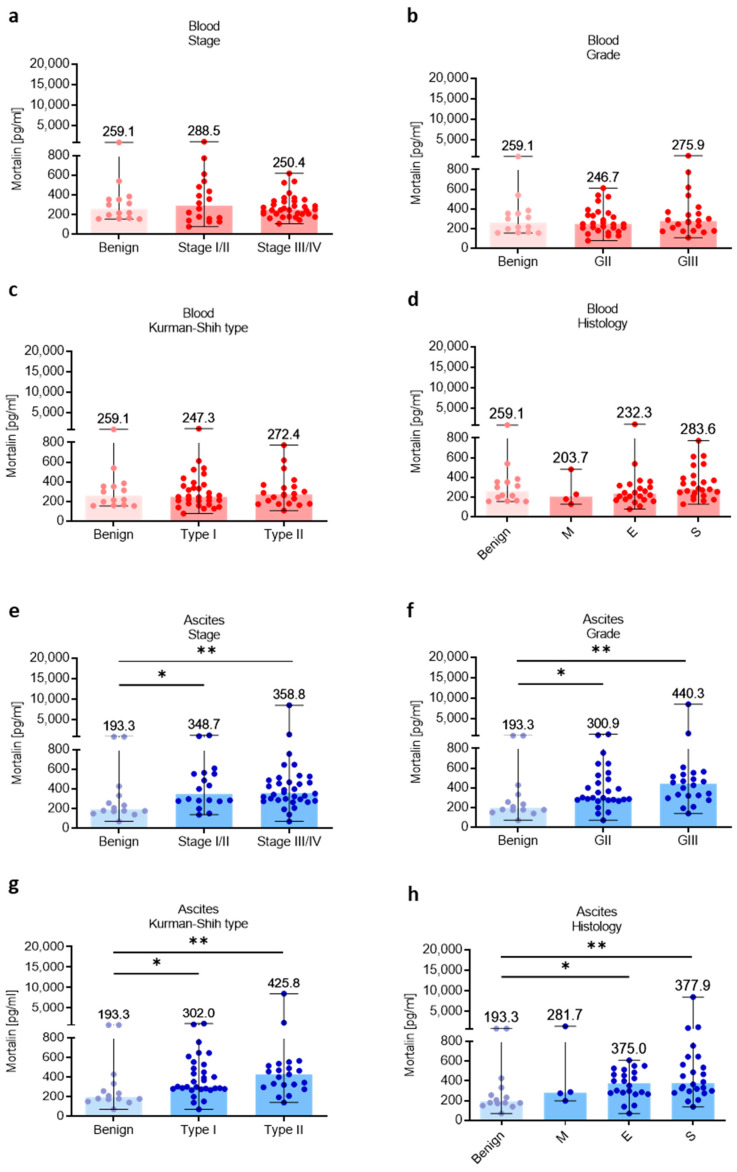
Mortalin profile in patients with different clinicopathologic characteristics. Mortalin levels in patients with benign ovarian tumors and ovarian cancer with different stage (**a**), grade (**b**), Kurman–Shih type (**c**), and histology type (**d**) in the blood plasma. Mortalin levels in patients with benign ovarian tumors and ovarian cancer with different stage (**e**), grade (**f**), Kurman–Shih type (**g**), and histology type (**h**) in the ascites fluid. Bars represent medians and ranges. * *p* < 0.05, ** *p* < 0.01. E—endometrioid OC; M—mucinous OC; S—serous OC.

Subsequently, the relationship of different clinicopathologic features of OC patients with levels of mortalin in the blood and ascites was investigated. The levels of blood-circulating mortalin were similar regardless of clinicopathological characteristic of ovarian tumors (*p* > 0.05, Figure 3a–d). In contrast, we observed higher abundance of mortalin in the ascites fluid in low (I/II) and advanced (III/IV) stage (*p* < 0.05 and *p* < 0.01, respectively, Figure 3e), grade II and III (*p* < 0.05 and *p* < 0.01, respectively, Figure 3f), type I and II (*p* < 0.05 and *p* < 0.01, respectively, Figure 3g), and endometrioid and serous histology types (*p* < 0.05 and *p* < 0.01, respectively, Figure 3h) of OC compared with benign ovarian tumors. It is well known that malignant ascites creates a cancer-driven immunosuppressive ecosystem which promotes OC metastases [30]. Our previous reports indicated M-MDSCs as the main population with immunosuppressive properties in OC patients [5,31]. Indeed, we observed significantly higher levels of immunosuppressive M-MDSCs in the ascites compared to blood in OC patients (*p* < 0.05, Figure S2a). Interestingly, when analyzing the relationship between local mortalin and myeloid cells, we showed that mortalin level had a positive correlation with M-MDSC in the ascites (R = 0.43, *p* = 0.04, Figure S2b). Next, we asked which cell types contribute more to the upregulation of mortalin. Using human serous OC scRNA-seq data (Figure S2c), we showed higher expression of *HSPA9* in tumor cells compared to tumor-infiltrating immune cells (i.e., myeloid cells and T cells). In concordance with this, we observed higher expression of mortalin protein in malignant tumor cells compared to tumor-infiltrating myeloid cells in HGSOC patients. (Figure S2d). Overall, this indicates that malignant tumor cells can be the major producers of soluble mortalin in the local environment. Most importantly, mortalin protein analysis of a matched cell line series derived from ascites or pleural effusions from two patients with HGSOC before and after development of clinical platinum resistance (PEO1 sensitive/PEO4 resistance and PEO14 sensitive/PEO23 resistance) identified significantly higher mortalin expression in platinum-resistant cells compared to platinum-sensitive cells (*p* < 0.003 and *p* < 0.04, respectively, Figure S1c), indicating mortalin as an one of the factors that can promote drug resistance in OC.

### 3.4. Mortalin Gene Expression Correlates with Cancer-Driven Gene Signatures and Is Associated with Worse Clinical Outcome

**Figure 4:**
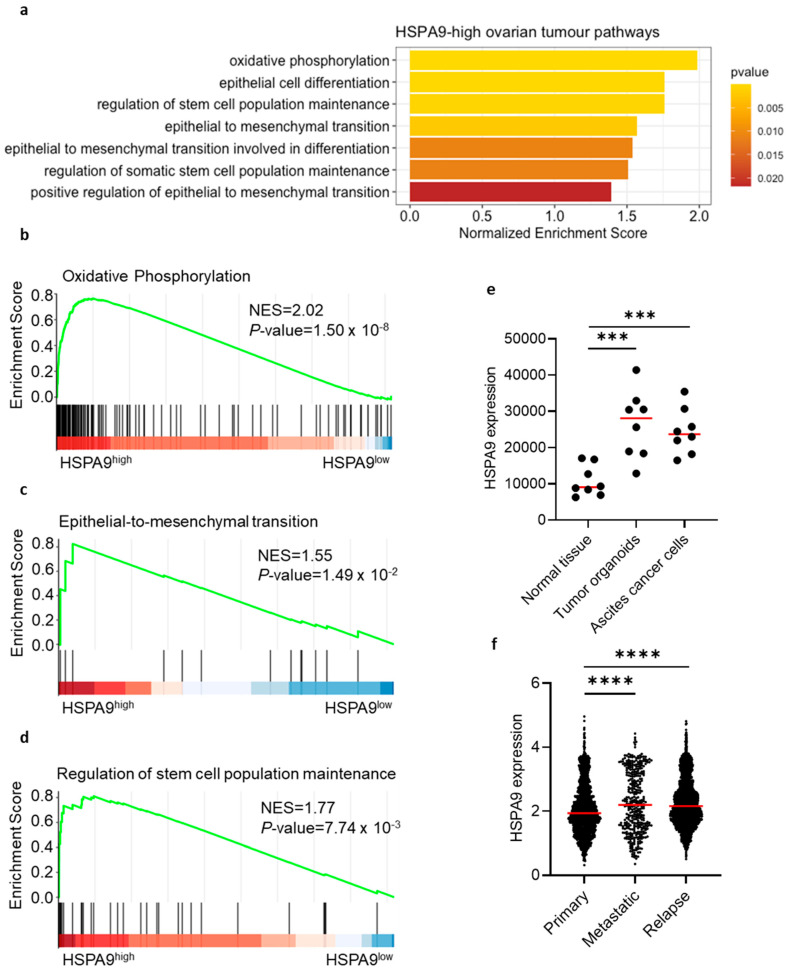
Mortalin gene expression correlates with cancer-related gene signatures in patients with ovarian cancer. Pathway enrichment using scRNA-seq in *HSPA9*-high ovarian tumors (**a**). Correlation analysis of mortalin with OXPHOS-related genes (**b**), EMT genes (**c**), and stemness-like genes (**d**). Mortalin gene expression of malignant fluids of serous OC, including tumor-derived organoids and malignant effusion cells (no cultured) paired with normal ovarian tissues (**e**). Mortalin gene expression in primary, metastatic, and relapse tumors in HGSOC (**f**). *p* < 0.001 ***; *p* < 0.0001 ****.

Next, we attempted to validate our observations that mortalin can promote OC pathogenesis. Using human serous OC scRNA-seq data, GSEA demonstrated enrichment of several cancer-related gene pathways in OC with high mortalin (*HSPA9*) expression (Figure 4a). These pathways included oxidative phosphorylation (OXPHOS) signaling (Figure 4b, EMT signaling (Figure 4c), and stemness maintenance (Figure 4d). In concordance with this, mortalin was correlated with OXPHOS-related genes (*CYCS, COX5B, COX8A, SDHD,* and *ATP5F1B*) (Figure S3a) and EMT stemness-like related genes (*STK3, STK4, PAX2, PAX8, CD24, SNAI1, SNAI2, MYC, TWIST2,* and *BMP7*) (Figure S3b). Therefore, mortalin expression can regulate functional potency and aggressiveness of OC cells. Indeed, mortalin gene expression was upregulated in the malignant fluid of serous OC, including tumor-derived organoids (cultured) and malignant effusion cells (no cultured) compared to normal ovarian tissues (*p* < 0.001, Figure 4e). Moreover, the mortalin gene was highly expressed in metastatic and relapse tumors compared to primary tumors in HGSOC patients (*p* < 0.0001, Figure 4f). Collectively, the results supported that mortalin can support worse clinical outcome.

### 3.5. High Tumor Mortalin Levels Predict Poor Prognosis

**Figure 5:**
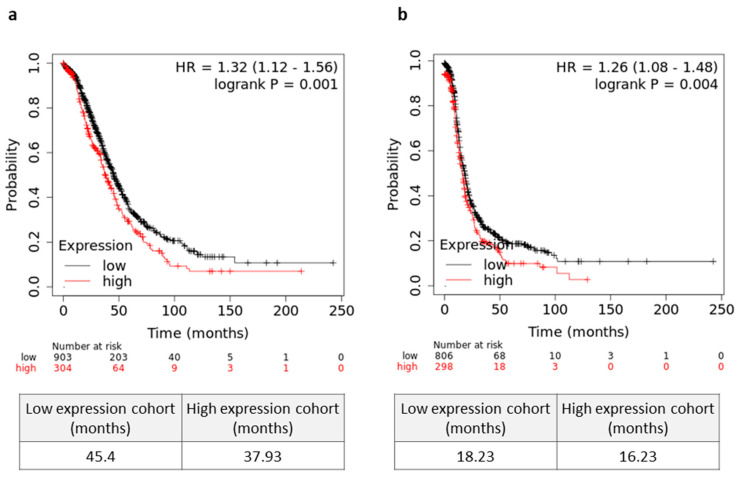
Prognostic relevance mortalin in serous ovarian cancer (OC) patients. (**a**) The overall survival (OS) (**a**) and progression free survival (PFS) (**b**) values of the OC patients with high/low levels of *HSPA9* are shown as Kaplan–Meier curves. Microarray datasets (online KM plotter database, JetSet best probe set) were used for mortalin gene expression.

Lastly, we asked whether mortalin can be an independent prognostic factor in serous OC patients. As shown in Figure 5a, patients with a high tumor mortalin gene expression levels showed significantly decreased OS (*p* = 0.001). Moreover, higher expression of mortalin was associated with worse PFS (*p* = 0.004) in OC patients (Figure 5b). In contrast, there was no significant change in OS in patients with different levels of soluble mortalin in the blood and peritoneal fluid (*p* > 0.05, Figure S24a,b, respectively).

## 4. Discussion

Among all gynecological malignancies, OC is the most lethal disease. Therefore, a better understanding of the malignant features of this disease is relevant. To our knowledge, this is the first study to examine the profile of mortalin in the peripheral and local tumor ecosystem in OC patients. Although previous studies examined mortalin within OC tumor tissue, results were inconclusive. Additionally, data of extracellular soluble mortalin in the blood and ascites of OC are lacking. In the present study, we evaluated parallel analysis of mortalin in the peripheral (blood plasma) and local (ascites fluid, tumor tissue) ecosystem and its clinical relevance in OC pathology.

In our study, we found significantly increased level of mortalin protein in ascites and tumor tissue in OC patients compared to control. These observations confirm a previous study which demonstrated elevated expression level of mortalin in OC [22]. In contrast, another research group revealed higher mortalin expression versus normal controls in 16/24 tumor tissues including bladder, brain, breast, colon, duodenum, fallopian tube, gallbladder, kidney, liver, pancreas, parotid, prostate, thymus, thyroid, ureter, and uterus neoplasms, and no significant change in mortalin level in 8/24 tumor tissues (including ovary, adrenal gland, lung, esophagus, rectum, stomach, testis, and lymphoma) [13]. Although we did not observe a higher level of soluble mortalin in the blood of OC patients compared to the control group, extracellular soluble blood mortalin was documented in colorectal cancer, and data showed a significantly higher level of mortalin in these patients compared to control [17,32]. Overall, this indicates that peripheral blood is not the primary source of soluble mortalin in OC patients. It is well known that mortalin drives cancer pathogenesis, while malignant ascites creates an immunosuppressive ecosystem, representing the main route of OC metastases. Indeed, we observed significantly higher levels of mortalin in local tumor ascites compared to peripheral blood in malignant OC.

To evaluate the clinical relevance of soluble mortalin in the blood and ascites, we integrated the above data with clinicopathologic features of OC patients. We observed similar levels of blood mortalin regardless of clinicopathologic characteristics of patients. Previous studies also demonstrated stage and grade disease-independent accumulation of serum mortalin concentration in patients with colorectal cancer [17,32]. Interestingly, our findings imply a higher level of ascites mortalin in OC patients with advanced stage (III/IV), high grade (GII/III), type I/II Kurman–Shih, endometrioid, and serous histology type compared with benign ovarian tumors. Our data led us to the conviction that high mortalin level in the ascites can be a characteristic feature of malignant disease and can play significant role in its peritoneal dissemination.

Function analysis of the conserved marker genes indicated that OC with a high level of tumor *HSPA9* was mainly related to OXPHOS pathways (e.g., CYCS, COX5B, COX8A, SDHD, and ATP5F1B) and EMT-stemness-like related pathways (e.g., STK3, STK4, PAX2, PAX8, CD24, SNAI1, SNAI2, MYC, TWIST2, and BMP7). It has been shown that OXPHOS is characteristic of OC stem cells (OCSCs), suggesting that OCSCs favor OXPHOS over glycolysis. Moreover, chemosensitive cells rely mainly on glycolysis, while chemoresistant cells have the ability to switch between glycolysis and OXPHOS [33]. Therefore, mortalin may be one of the factors promoting OC chemoresistance. Indeed, analysis of mortalin protein in a matched cell line series from two patients with HGSOC before and after development of clinical platinum resistance (PEO1/PEO4, PEO14/PEO23) showed upregulation of this protein in resistant OC cells. It has been demonstrated that expression of the OXPHOS pathway was elevated in resistant cell lines [27], and mortalin upregulation can be associated with resistance of OC cells to cisplatin [14]. Indeed, a chemosensitive cancer cell line (PEO1) displayed a glycolytic phenotype, while its chemoresistant counterpart (PEO4) exhibited a high metabolically active phenotype with the ability to switch between OXPHOS and glycolysis [34]. Importantly, mortalin was highly expressed in metastatic and relapse HGSOC compared to primary tumors and correlated with immunosuppressive M-MDSCs. Overall, this may indicate that a certain subpopulation of tumor cells may exist and evade chemotherapy, perhaps with the assistance of mortalin and other immune cells (e.g., M-MDSCs), migrating out of the primary site to initiate relapse OC tumors. Further study will be needed to validate this concept. Nevertheless, previous data showed that downregulation of mortalin expression was associated with a reduction in OC cell proliferation, colony formation, and migration/invasion, which confirms that mortalin can promote the development and progression of OC [35]. In concordance with this, we revealed that high mortalin gene expression level is negatively correlated with OS and PFS in OC patients, indicating the prognostic value of this biomarker in OC disease.

Of note, treatment of cancer cells with mortalin short hairpin (sh)RNA or inhibitors reverted the drug resistance of cells and suppressed their migration and invasion properties [21]. Intriguingly, extracellular Hsp70 forms an activation complex with different Hsps, including Hsp90α, Hsp70/Hsp90 organizing protein (Hop), and Hsp40, which together enhance the invasion and migration of the breast cancer cells via the upregulation of metalloproteinase-2 (MMP2). Furthermore, previous findings revealed that mortalin enhances the resistance of cancer cells to complement-dependent cytotoxicity (CDC) and can, thus, promote tumor escape and attenuate immunotherapy efficacy [18]. Taking into consideration that only a small percentage of OC patients (~15%) respond to immunotherapy [36], our findings should be taken into consideration in cancer immunomonitoring and design of future (immuno)therapeutic trials.

## 5. Conclusions

Firstly, mortalin is highly upregulated in local ecosystems, i.e., ascites and tumor tissues in OC patients compared to control groups. Secondly, high expression of local tumor mortalin is associated with cancer-driven signaling pathways (i.e., OXPHOS and EMT/stemness-like signaling) and worse clinical outcome. Thirdly, high tumor mortalin level is associated with bad OS and PFS.

To sum up, our current findings are important to investigators in the OC field and those who are working on development of new biomarkers and (immuno)therapies.

## Figures and Tables

**Table 1:** Patient characteristics.

Category	Blood	Ascites
Healthy women, N	28	NA
Benign, N	14	14
Ovarian cancer, N	50	50
Age at diagnosis, mean (min–max)		
Healthy women	40.58 (25–66)	NA
Benign	49.71 (23–86)	49.71 (23–86)
Ovarian cancer	58.56 (20–86)	58.56 (20–86)
Stage, N		
Early I/II	18 (36.1)	18 (36.1)
Advanced III/IV	32 (63.9)	32. (63.9)
Grade, N		
II	28	28
III	22	22
Kurman–Shih type, N		
I	30	30
II	20	20
Histology, N		
Mucinous	4	4
Endometrioid	22	22
Serous	24	24

NA—not applicable.

## Data Availability

The datasets used in the current study are available from the corresponding author on reasonable request.

## Supplementary Materials

The following supporting information can be downloaded at https://www.mdpi.com/article/10.3390/cells12050701/s1.
