# Supplementary material for: Clinical Relevance of Mortalin in Ovarian Cancer Patients
Source: Cells. 2023 Feb 23;12(5):701. doi: 10.3390/cells12050701 (PMC10000941; doi:10.3390/cells12050701)
Supplement: Supplementary file 1 [file cells-12-00701-s001.zip › cells-2217378-supplementary.pdf]

cells MDPI

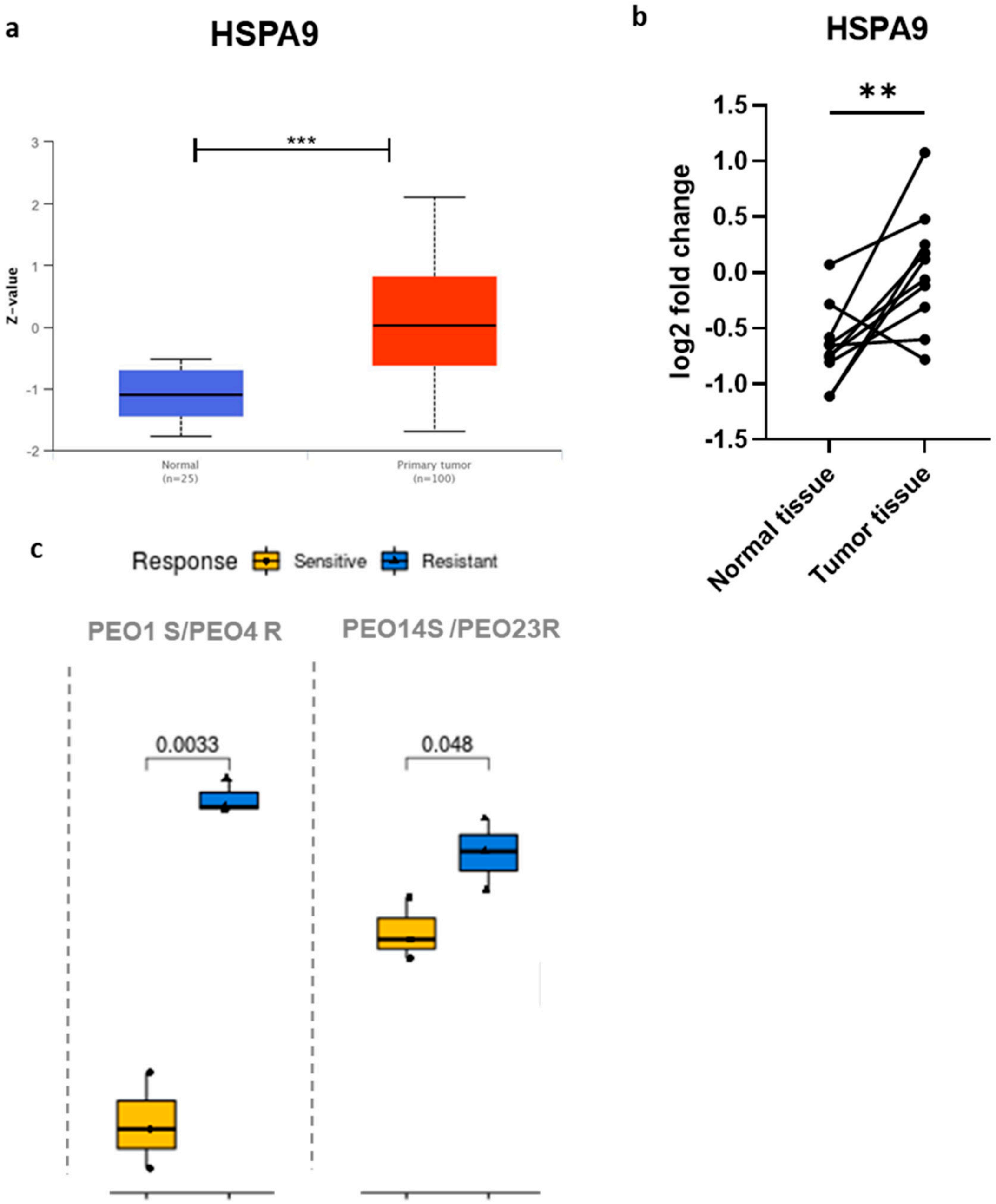

Figure S1. Mortalin protein expression in normal and ovarian tumor tissue and cells. Analysis performed using UALCAN portal with data of ovarian cancer (OC) and normal tissues from Clinical Proteomic Tumor Analysis Consortium (CPTAC), available at http://ualcan.path.uab.edu/. Z-values represent standard deviations from the median across samples for the given cancer type of OC. *** p-value < 0.001. Comparison of mortalin protein expression in normal tissues and OC primary tumors **(a)**. Comparison of mortalin protein expression in 10 normal tissue samples which were matched with tumor samples from the same HGSOC patient **(b)**. Comparison of mortalin protein expression in matched cell-line series from two patients with HGSOC before and after development of clinical platinum resistance (PEO1 sensitive/PEO4 resistance, PEO14 sensitive/PEO23 resistance) **(c)**.

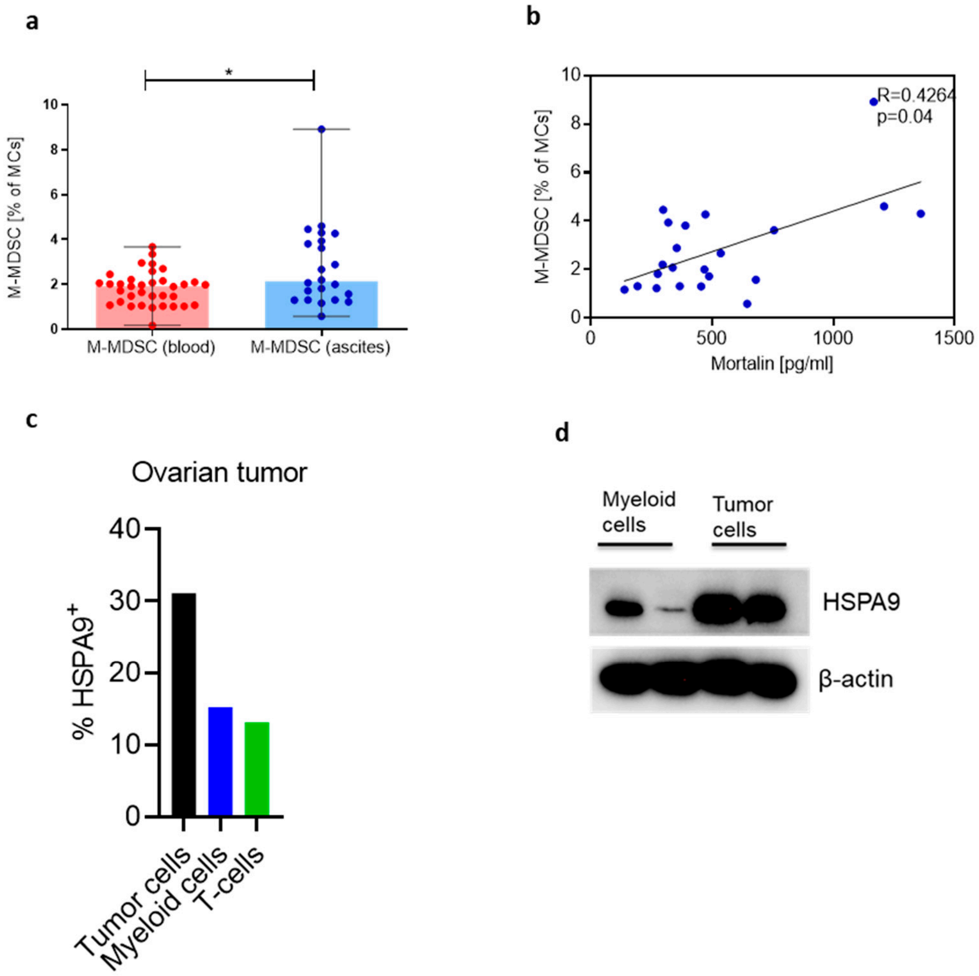

Figure S2. Analysis of monocytic myeloid-derived suppressor cells (M-MDSCs) and mortalin expression in ovarian cancer (OC). Mononuclear cells (MCs) from the blood and ascites of OC patients were analyzed. Analysis was performed using flow cytometry. MCs were stained for M-MDSCs using fluorochrome-labeled monoclonal antibodies (mAb) against HLA-DR and CD14. Bars represent medians and ranges. *$p<0.05$ **(a)**. The correlation between the abundance of M-MDSCs and mortalin in the ascites from OC patients **(b)**. Mortalin gene expression analysis of different population of cells using scRNA-seq data (accession number GSE211956, n=8 HGSOC patients) **(c).** Mortalin protein expression analysis of tumor cells and tumor-infiltraing myeloid cells (n=2 HGSOC patients).

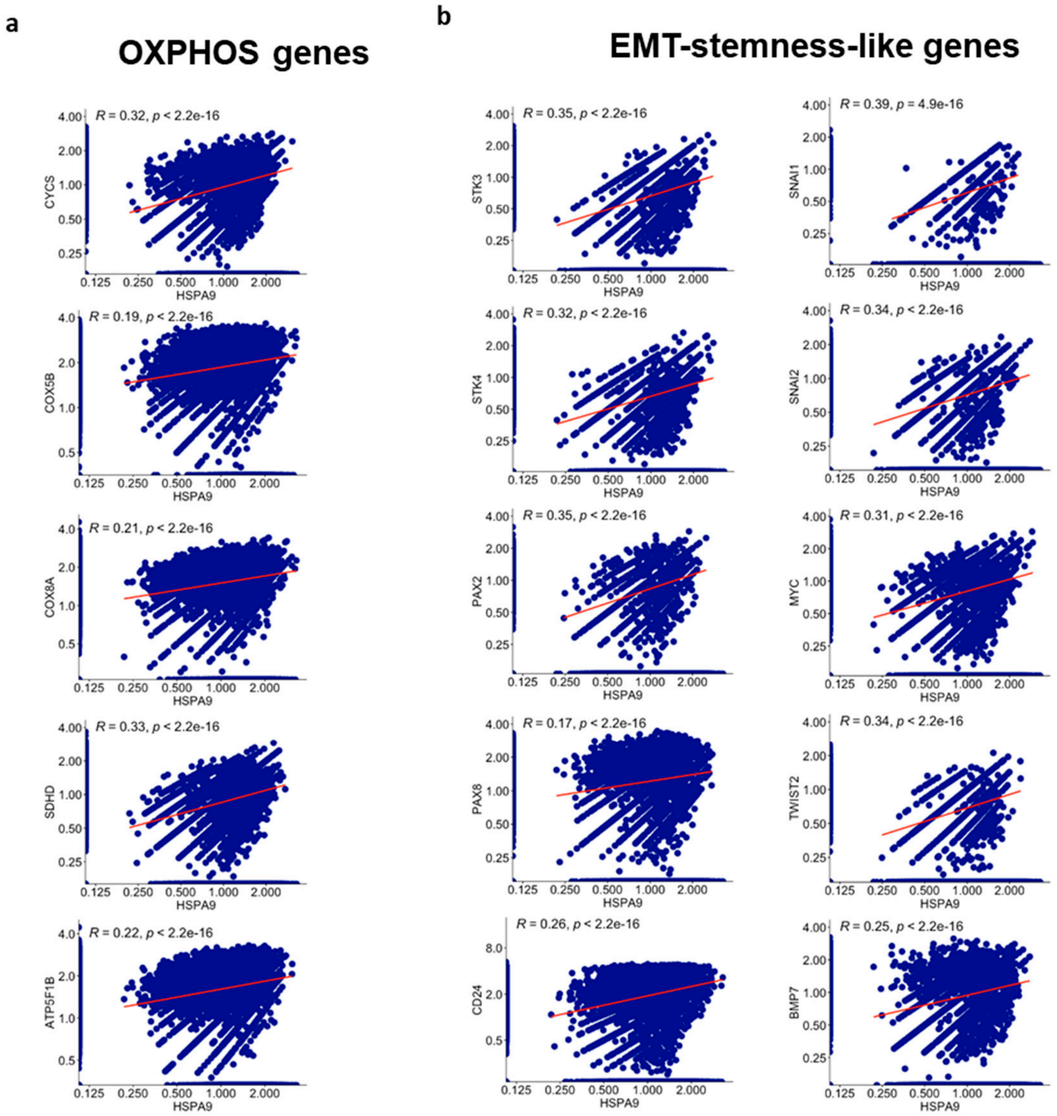

Figure S3. Tumor mortalin gene expression correlates with cancer-related gene signatures in patients with high grade serous ovarian cancer (HGSOC). Spearman correlation analysis of tumor mortalin with OXPHOS-related genes (a) and EMT-stemness-like genes (b). scRNA-seq data were obtained from the Gene Expression Omnibus database (accession number GSE211956, n=8 HGSOC patients).

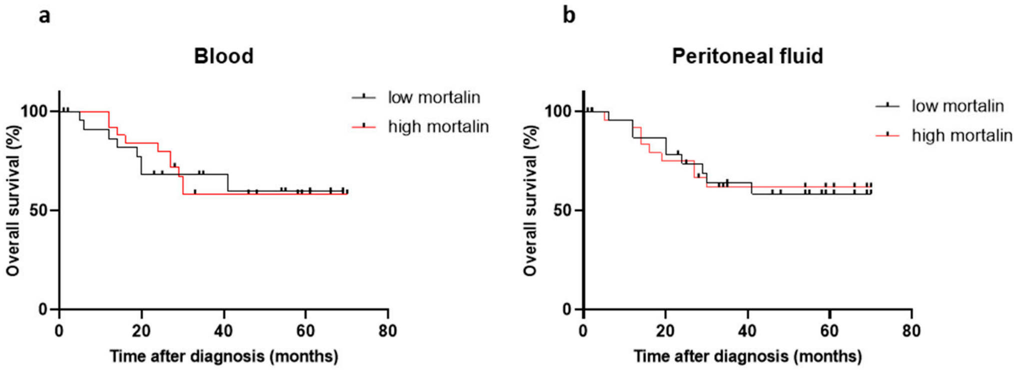

Figure S4. Prognostic relevance of soluble mortalin in ovarian cancer (OC) patients. **(a)** The overall survival (OS) values of the OC patients with low/high levels of mortalin in the blood **(a)** and peritoneal fluid **(b)** are shown as Kaplan-Meier curves.
